# Supplementary material for: Scorpionism in Brazil: Space-time approach and risk areas in 2012 to 2024
Source: PLoS Negl Trop Dis. 2025 Oct 15;19(10):e0013602. doi: 10.1371/journal.pntd.0013602 (PMC12561935; doi:10.1371/journal.pntd.0013602)
Supplement: Attachment — Submitted filename: Answers.docx [file pntd.0013602.s003.docx]

**Response to Reviewers**

We would like to sincerely thank the editor and the reviewers for their valuable time, thoughtful comments, and constructive suggestions. Their insights have greatly contributed to improving the clarity, quality, and overall impact of our study. We truly appreciate their dedication to enhancing the scientific value of our work.

Reviewer No. 3: In Tables 1 and 2, the differences between the two incidence indicators and between the variables “cases” and “n” are unclear. In addition, the table layout would be clearer if the first column contained the indicators, followed by three more columns for presenting the data: one for women, one for men, and one for general data.

Answer: The “cases” and “n” were adjusted as recommended. The standardization of the tables was maintained according to the last update, and it was not possible to change the current format because it involves too many variables for observation (analysis by sex, age groups and other indicators).

In the paragraphs beginning on lines 140 and 148, Table 1 should be referenced.

Answer: Adjustment made.

In Table 3, “T-test (mean values)” should be removed from the matrix row, as it creates confusion regarding the data presented. It is also unnecessary to include the footnote “* not significant.”

Answer: Adjustment made.

Reviewer No. 1: The conclusions are relevant. However, some points were not addressed, such as the high lethality in the North region and the significant risk of accidents in the states of Sergipe/Alagoas.

Answer: Adjustment made. Information on lethality in the northern region and aspects of the northeastern states has been included in the text.

In the discussion, it would be important to elaborate on the differences in lethality between regions, since the North has approximately double the percentage for men and women (Table 2), which may be related to North-South inequalities in Brazil (the North is more economically disadvantaged).

Answer: Adjustment made. Information on lethality in the northern region and aspects of the northeastern states has been included in the text.

Reviewer #3: The bibliography is somewhat outdated; only 15 of the 48 references are from the last five years, and none are from last year, despite the existing scientific output in the field (40 references with “Scorpion Stings” [Mesh] in PubMed in the last year). The bibliographic references are not listed in alphabetical order by author.

Answer: Some more up-to-date references have been added to the discussion as suggested. Most of the older mentions are considered more classic literature and have been kept together.

Although it is part of the study's objectives, it is noteworthy that the Introduction does not mention the relationship between the spread of scorpionism and climate change, which should have been mentioned in this section. An interesting work to consult: Lira, AFdA, Badillo-Montaño, R., Lira-Noriega, A., and de Albuquerque, CMR (2020), Potential distribution patterns of scorpions in northeastern Brazil under future climate change scenarios. Ecologia Austral, 45: 215-228.

Answer: Adjustment made. A small approach was made in the introduction to mention the aspects of climate change.

The first important point is that I believe it would be beneficial for the authors to extend the study period and include available data from 2022, 2023, and 2024. This would allow for an updated version that could serve as a reference for future studies.

Answer: The data was updated as requested, including the years 2022, 2023 and 2024, and the analyses were reproduced again with some changes to the results.

The second important point concerns statistical analyses using the t-test. Does your response variable follow a normal distribution? I ask this because I believe it is unlikely that the variables used have a normal distribution, which means that comparing means may not be the best strategy.

Answer: The data was presented using the t-test of two means (with different or equivalent variances, depending on the F-test) as it is a more exploratory test and allows for more hypotheses on the subject. New, more comparative studies using statistical modeling are being developed based on these results so that we can have a more solid basis for comparison.

Lines 91-95 and 96-98: The authors should explain in more detail what Local Empirical Bayesian is, what type of criterion I refers to, which version of R was used, and whether any specific package was applied. In addition, what is the retrospective Poisson model? Without this information, someone unfamiliar with the subject will have difficulty replicating the study.

Answer: Adjustment made.

Line 115: The authors should clarify where the variables were obtained and at what resolution. There is a lack of information necessary for replication. Were the values obtained from the municipality's centroid? Was an average used? What software was used to process the variables?

Answer: The adjustments are located on lines 134-137. "The variables were obtained from freely accessible information systems. The environmental and climatic variables were taken from MapBiomas and WorldClim and the socio-demographic data was collected from IBGE systems."

Figure 6: A better explanation is needed. The caption does not even indicate what panel B represents.

Answer: Adjustment made.

I believe there should be a discussion about lethality in northern Brazil, as the data presented in Table 2 clearly show that it is almost double that of other regions.

Answer: Adjustment made. Information on fatalities in the northern region has been inserted into the text.

Looking at the map in Figure 3, Sergipe/Alagoas also appears as a high-risk region. However, throughout the text, this is not mentioned, and the focus is only on Bahia. Why?

Answer: Text adjusted to compose this information on the northeast region. Line 209-212

The text should undergo a complete revision to refine the use of English, ensuring the accuracy of scientific terminology. Grammatical errors should be corrected, such as the incorrect use of singular verbs with plural nouns (for example, “Data” in line 83 should use a plural verb). The use of non-standard or unusual word combinations should be revised to improve readability and maintain scientific accuracy.

Answer: The English text has been re-evaluated to maintain acceptable quality standards.

Although the introduction provides an adequate overview of scorpionism in Brazil, a broader discussion of its global impact would be useful. The inclusion of a brief comparison with other endemic regions would help to contextualize the importance of scorpionism in Brazil in relation to global trends.

Answer: Adjustments made. Line 56-59

The article lacks sufficient detail on the composition of the venom and the clinical effects of different species of scorpions. A brief discussion of interspecific differences in venom toxicity, its physiological impact, and clinical manifestations would strengthen the scientific basis of the study.

Answer: Adjustments made. Some points have been added on the factor mentioned.

The study does not clarify whether confounding factors, such as socioeconomic status and access to medical care, were controlled for in the analysis.

Answer: As this was an exploratory study, confounding factors were not dealt with, and an analysis using statistical modeling is planned for future studies on associations with accidents and other indicators of epidemiological interest

The abstract should be structured with subheadings (e.g., Background, Methods, Results, and Conclusion) to improve clarity and readability.

Answer: Adjustment made.

The recognition of financing should be moved to a separate section rather than being included in the methodology.

Answer: Adjustment made. Line 139-141.

The discussion section should be reorganized for better logical flow. Redundant information should be removed or consolidated, such as the multiple mentions of antivenom treatment, which should be grouped into a dedicated section.

Answer: Adjustments made. New points have been added and the discussion is aligned to answer, respectively: information on risk groups, severity factors, case growth, environmental aspects, survival and biological control, risk areas in Brazil, aspects of the northern region, lethality of accidents, importance of geographical epidemiology, surveillance and statistical analysis, adaptability of services, strengths and limitations.

The role of biological control methods (e.g., chickens, cururu toads) is an interesting aspect, but currently lacks sufficient depth. Incorporating evidence-based discussions, such as documented studies on their effectiveness, would enrich this section.

Answer: The information on biological control was kept and new elements were added to the discussion, so as not to centralize discussions in such sensitive niches with little scientific evidence.

The only suggestion I would make is to include a summary of your results in the discussion. This will help us remember all the results presented in the article. So consider it as the first paragraph of the discussion.

Answer: Adjustment made. Line 276-280.
